# Clinical characteristics and cytological changes in mucinous obstruction diagnosed by dacryoendoscopy

**DOI:** 10.1038/s41598-024-59580-9

**Published:** 2024-04-17

**Authors:** Doah Kim, Helen Lew

**Affiliations:** Department of Ophthalmology, CHA Bundang Medical Center, CHA University, #59 Yatap-Ro, Bundang-Gu, Seongnam, 13496 Republic of Korea

**Keywords:** Lacrimal apparatus diseases, Eye abnormalities

## Abstract

To analyze the clinical characteristics of mucinous obstruction diagnosed by dacryoendoscopy and compared the cytological changes with membranous obstruction using a modified liquid-based thin prep cytology method. A retrospective chart review was conducted on 53 eyes of 51 patients with mucus obstruction based on dacryoendoscopic findings from January 2022 to October 2022. Liquid-based thin-prep cytology was performed by irrigating the inside of the nasolacrimal drainage system with saline during dacryoendoscopy-guided silicone tube intubation. Pathological findings were analyzed through a comparison of mucinous obstruction with membranous obstruction as determined by dacryoendoscopic findings. The modified liquid-based thin prep cytology technique had a higher cytology detection rate across all cases. Mucinous obstruction exhibited a significantly higher number of successful canalicular irrigation test cases compared to membranous obstruction. In mucinous obstruction, epithelial squamous cells were more frequently detected in pre-sac obstruction, whereas columnar epithelial cells were predominant in post-sac obstruction. Inflammatory cells showed a stronger correlation with primary change and post-sac obstruction. Bacterial colonies were observed exclusively in cases of mucinous obstruction. The use of a modified liquid-based thin prep cytology method enables the examination of histopathological changes in the lacrimal passage in primary acquired nasolacrimal duct obstruction (PANDO), particularly in cases of mucinous obstruction, without the need for invasive biopsies. These findings enhance the understanding of the etiopathogenesis of mucinous obstruction, complementing knowledge of membranous obstruction in PANDO.

## Introduction

Primary acquired nasolacrimal duct obstruction (PANDO) is a prevalent disorder affecting the nasolacrimal duct (NLD) drainage system in adults.^1^ The underlying cause of PANDO is believed to involve inflammation and fibrosis within the lacrimal drainage system (LDS), although the precise etiology remains uncertain.^2^ Several factors have been proposed to contribute to the development of nasolacrimal duct obstruction (NLDO), such as anatomical variation, vascular changes, autonomic dysfunction, sino-nasal factors, hormonal influences, tear-related factors, and the lacriome.^3^

Dacryoendoscopy has emerged as a valuable technique for direct visualization of the LDS, facilitating the identification of various obstruction causes such as stenosis, mucoceles, and dacryoliths.^4–6^ Based on dacryoendoscopic confirmation of the obstructive type, PANDO can be categorized into two main types: secretory or nonstructural changes involving mucus, granuloma, and stones, and structural changes such as fibrotic membranes or stenosis.^7^ Nonstructural epiphora, characterized by secretory changes, is referred to as “functional NLDO” (FNLDO).^8^ FNLDO patients experience a similar severity of epiphora as those with NLDO, despite having patent but dysfunctional NLD drainage.^8,9^ While numerous studies have focused on the treatment of FNLDO, few have analyzed cytological levels or compared them with anatomical NLDO. Previously, we analyzed the cytology of PANDO using a liquid-based thin prep cytology method, demonstrating its potential as a noninvasive approach to examining histopathological changes in PANDO.^10^.

In the present study, we analyzed the clinical characteristics of mucinous obstruction diagnosed through dacryoendoscopy and investigated the clinical features and cytological changes in comparison to membranous obstruction using a newly modified liquid-based thin prep cytology method. By examining this, we seek to enhance understanding of the pathogenesis and differences between mucinous and membranous obstructions in PANDO.

## Methods

### Patients

**Figure 1:**
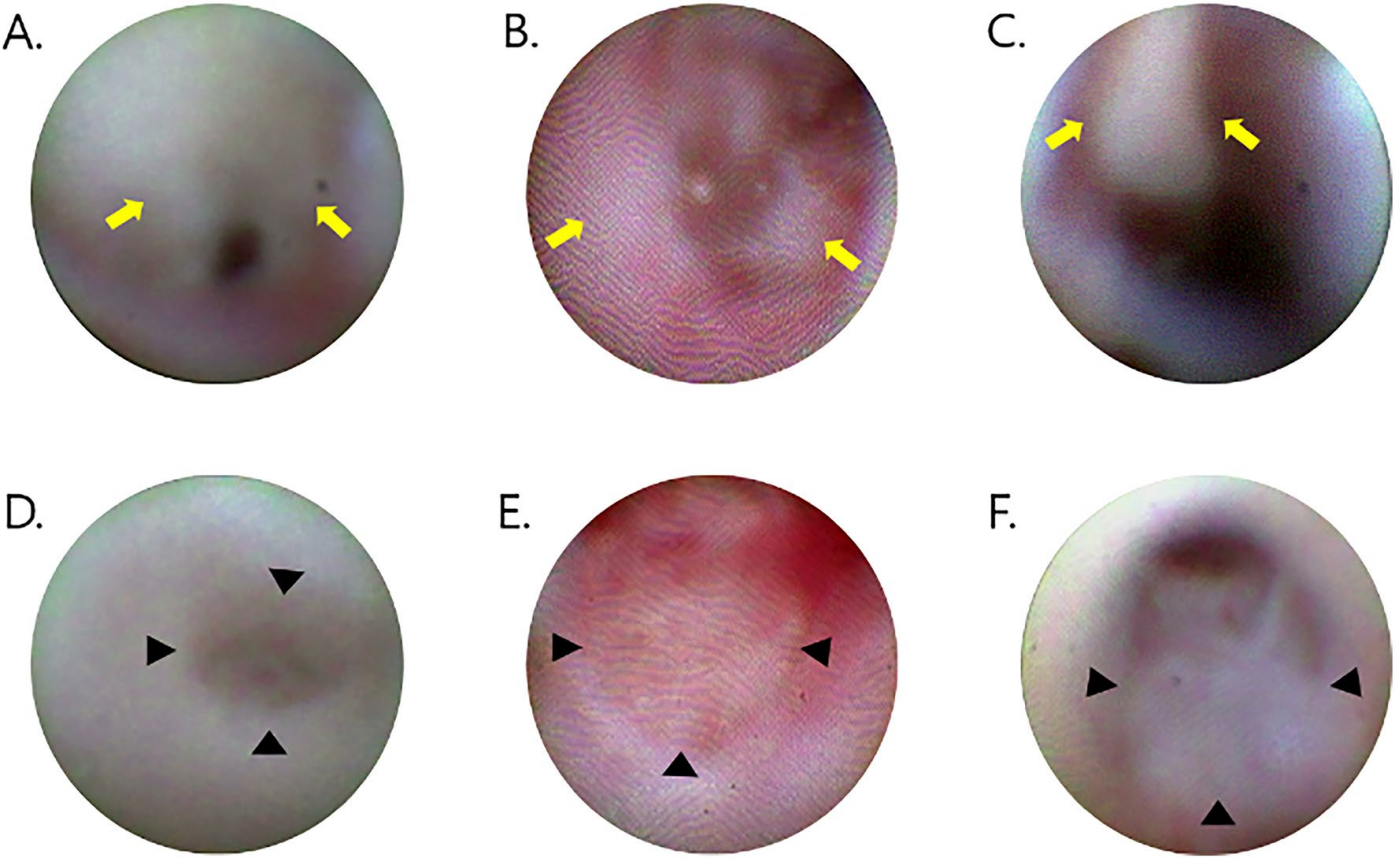
Dacryoendoscopic images of mucinous obstruction according to the level of obstruction in lacrimal drainage system (**A**: canaliculus, **B**: lacrimal sac, **C**: nasolacrimal duct, yellow arrow: mucus) and membranous obstruction according to the level of obstruction in lacrimal drainage system (**D**: canaliculus, **E**: lacrimal sac, **F**: nasolacrimal duct, black arrowhead: membrane).

The dacryoendoscopic findings of 123 eyes of 108 patients diagnosed with PANDO at Bundang Cha Hospital from January 2022 to October 2022 were reviewed. From these, the medical records of 51 patients (53 eyes) were analyzed retrospectively, including 22 patients (22 eyes) with membranous obstruction and 29 patients (31 eyes) with mucinous obstruction. The type of obstruction was confirmed through dacryoendoscopic findings obtained during transcanalicular dacryoplasty (Fig. 1). In cases where membranous obstruction and mucinous obstruction were observed to be mixed on dacryoendoscopic view, classification was determined by an experienced surgeon based on the predominant feature. Patients with conditions that could potentially affect the detection of inflammatory cells, such as autoimmune diseases, thyroid dysfunction, or the use of immunosuppressive drugs, were excluded. Clinical histories, duration of epiphora, tear meniscus height (TMH), canaliculus irrigation tests, and dacryocystography (DCG) were examined. DCG findings were classified as follows: Primary obstruction primarily involves structural changes within the nasolacrimal duct itself, ranging from simple narrowing to complete blockage, hindering the normal flow of tears. Conversely, secondary obstruction is characterized by features such as beading along the duct or dilation of the lacrimal sac. Surgical success was defined as an MUNK score of less than 1 point and lacrimal meniscus height (LMH) of less than 300 μm after silicone tube removal.

### Surgical technique and specimen collection

Preoperatively, a sterile cotton-tipped swab was gently inserted through a nostril and positioned under the inferior turbinate. Then it was rotated to collect secretions before being removed. The specimen was sent to the Department of Laboratory Medicine for nasal bacterial culture and further analysis. Culture negativity was defined as the absence of bacterial growth within 4 weeks.

**Figure 2:**
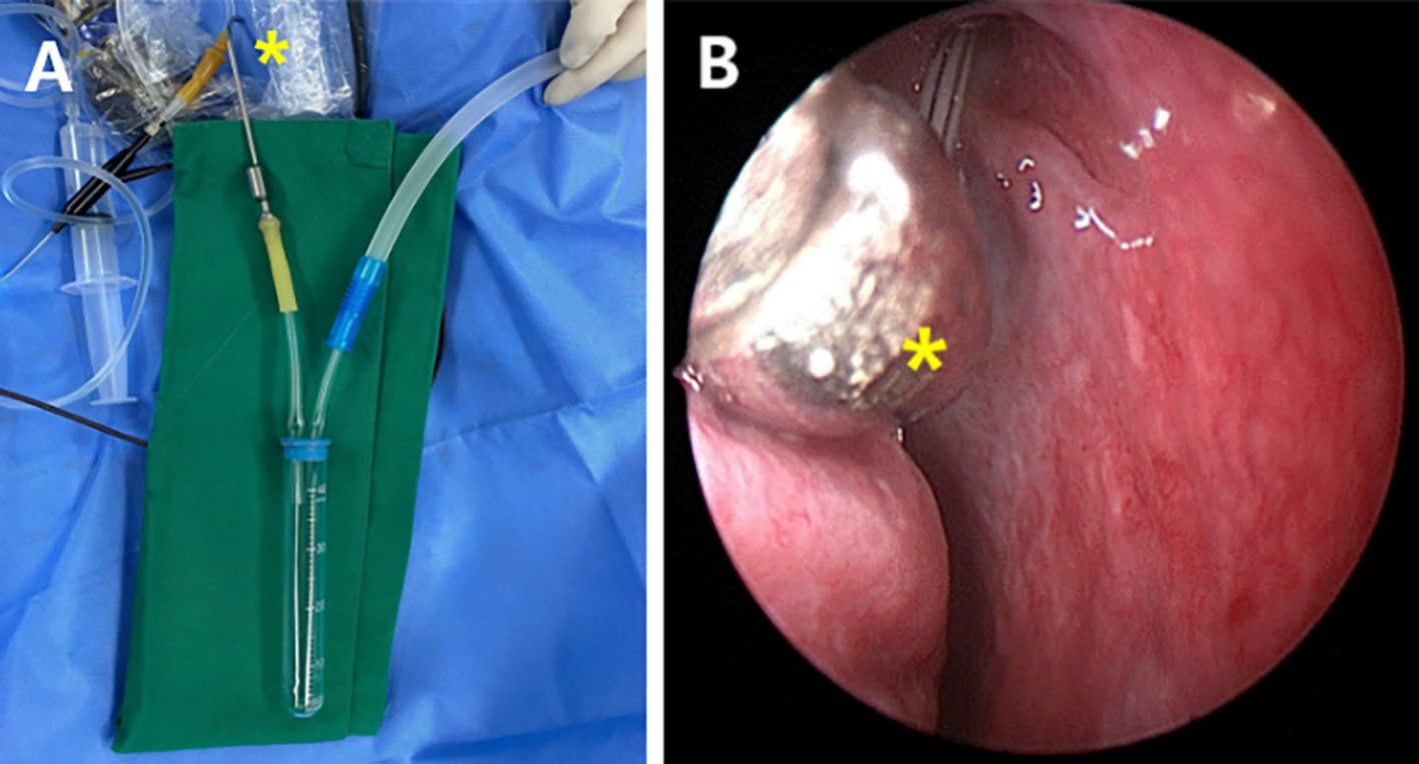
The specimen collection method of liquid-based thin prep cytology of lacrimal drainage system during transcanalicular dacryoplasty using sheath-guided dacryoendoscopy. (**A**) A specimen trap with a suction bottle connected to one side and a suction tip connected to the other side. (**B**) Suction tip located at the inferior meatus and collecting the sample that comes out after irrigation through lacrimal drainage system during recanalization. (Yellow asterisk *: Suction tip connected with specimen trap).

Transcanalicular dacryoplasty using sheath-guided dacryoendoscopy (RUIDO Fiberscope, Fiber Tech, Tokyo, Japan) was performed, as previously described.^10^ As a new addition to the technique, a specimen trap (DONGWHA PANDA, Incheon, Republic of Korea) was used to collect cells that remained in the sheath after passing through the LDS. One side of the specimen trap was connected to the suction line, while the other side was connected to the suction tip. The suction tip was positioned in front of the inferior meatus, and continuous irrigation with 3 mL of saline was performed through the sheath on the tip of the dacryoendoscope (Fig. 2). The collected specimens were placed in sterile bottles and sent directly to the Department of Pathology for cell analysis. All surgical procedures were performed by a single surgeon (H.L.).

Based on the dacryoendoscopic findings, we classified subjects into the mucinous type and membranous type. The mucinous type was defined as the presence of soft, whitish mucinous debris filling the inside of the LDS, while the membranous type was defined as the presence of a dense, membrane-like structural lesion occupying the LDS lumen.

### Statistical analysis

Statistical analyses were conducted using SPSS ver. 26.0 (IBM, Armonk, NY, USA). The Mann–Whitney test was used to compare nonparametric groups. A paired t-test was performed to compare values before and after surgery. A p-value less than 0.05 was considered statistically significant.

### Ethics approval and informed consent

All study participants provided informed consent before participation. The study was approved by the Institutional Review Board of Seongnam CHA Bundang CHA Hospital and adhered to the principles outlined in the Declaration of Helsinki (CHAMC 2023–08-060).

## Results

### Demographics and clinical characteristics

The study examined 53 eyes: 31 eyes with mucinous obstruction and 22 with membranous obstruction. There were no significant differences between mucinous and membranous obstruction in terms of the average age, sex distribution, duration of epiphora, allergy history, previous sexually transmitted infection, and TMH. However, a greater proportion of cases passed the preoperative canaliculus irrigation test in mucinous obstruction (45.2%) compared to membranous obstruction (13.6%) (p = 0.015). The preoperative DCG findings did not show any differences in the level or pattern of obstruction between the two groups. The overall surgical success rate was 91.3%, with success rates of 93.1% in mucinous obstruction and 88.2% in membranous obstruction (Table 1).

**Table 1:** Clinical characteristics and demography in the patients with mucinous obstruction and membranous obstruction on dacryoendoscopy.

	Mucinous obstruction (N = 31)	Membranous obstruction (N = 22)	Total (N = 53)	*p*-value
Age (years)	60.0 ± 12.9	59.5 ± 11.9	59.8 ± 12.4	0.874
Sex (male: female) (%)	13: 18 (41.9: 58.1)	5: 17 (22.7: 77.3)	18: 35 (34.0: 66.0)	0.122
Epiphora duration (months)	18.1 ± 8.5	21.5 ± 9.0	19.8 ± 6.8	0.482
Allergy history (%)	10 (32.3)	4 (18.2)	14 (26.4)	0.236
Previous STI history (%)	7 (22.6)	1 (4.5)	8 (15.1)	0.074
Tear meniscus height (um)	383.0 ± 224.8	386.9 ± 189.2	384.6 ± 208.8	0.891
Laterality (right: left)	14: 17	13: 9	27: 26	0.236
Preoperative irrigation test				
Passed: Not-passed (%)	14: 17 (45.2: 54.8)	3: 19 (13.6: 86.4)	17: 36 (32.1: 67.9)	0.015*
Preoperative DCG				
Type of obstruction Primary: Secondary (%)	21: 10 (67.7: 32.3)	11: 11 (50.0: 50.0)	32: 21 (60.4: 39.6)	0.123
Level of obstruction Pre-sac: Sac & post-sac (%)	23: 8 (74.2: 25.8)	8: 14 (36.4: 63.6)	31: 22 (58.5: 41.5)	0.197
Success rate (%)	93.1	88.2	91.3	0.663

*N* number, *STI* silicone tube intubation, *DCG* dacryocystography.

*p-value < 0.05.

### Bacterial culture of the nasal mucosa

**Figure 3:**
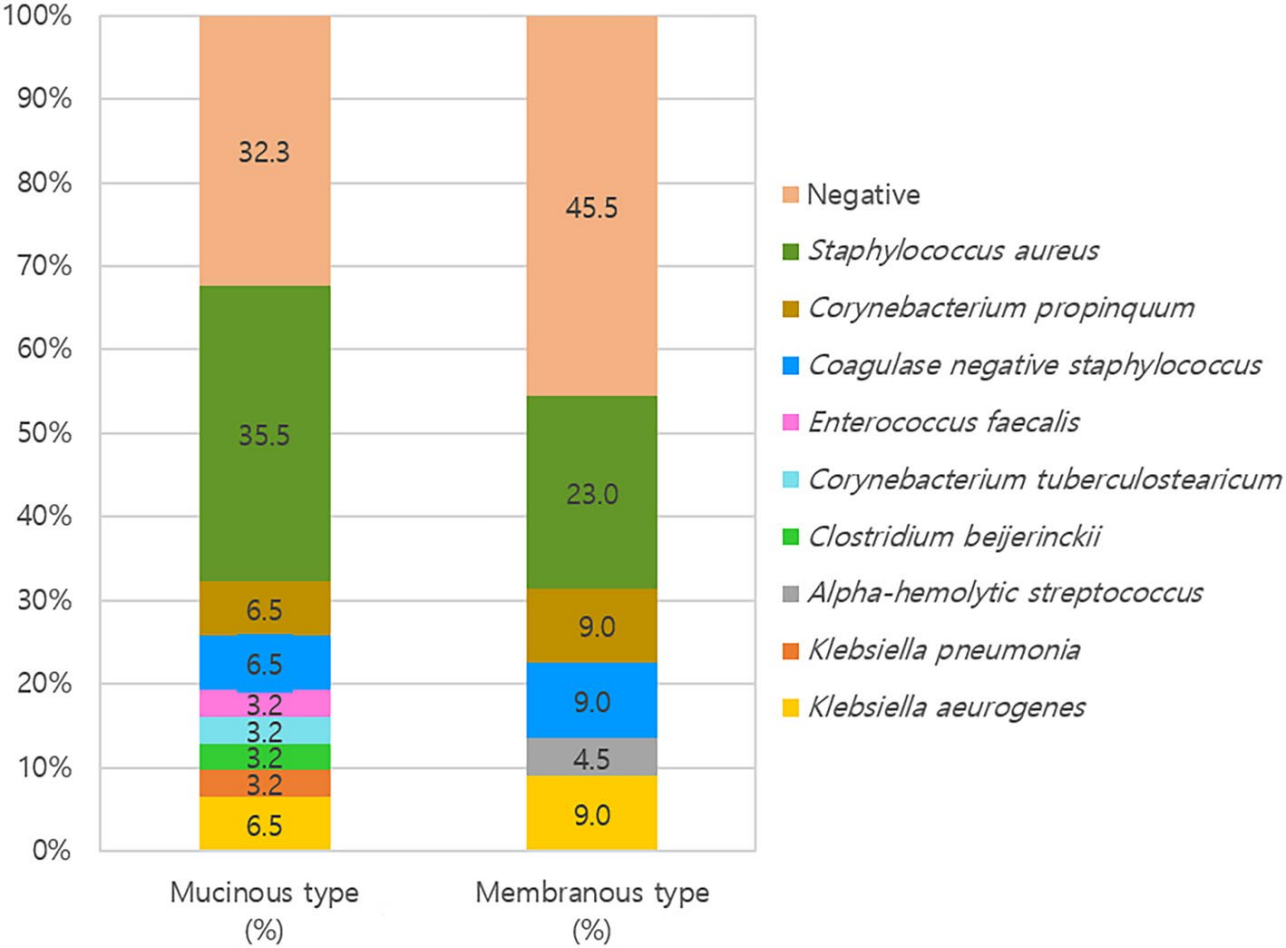
Comparison of bacterial species identified in preoperative bacterial culture using nasal swab in mucinous obstruction and membranous obstruction.

The preoperative bacterial detection rates were 67.7% (21/31) for mucinous obstruction and 54.5% (12/22) for membranous obstruction, and there were no significant differences between the two groups (p = 0.333). In both groups, *Staphylococcus aureus* was the most commonly detected bacterium (mucinous obstruction vs. membranous obstruction, 35.5% vs. 23.0%) followed *by coagulase-negative Staphylococcus* (6.5%), *Corynebacterium propinquum* (6.5%), *Klebsiella aerogenes* (6.5%), *Enterococcus faecalis* (3.2%), *Corynebacterium tuberculostearicum* (3.2%), *Clostridium beijerinckii* (3.2%), and *Klebsiella pneumonia* (3.2%) in mucinous obstruction, and by *C. propinquum* (9.0%), *coagulase-negative Staphylococcus* (9.0%), *K. aerogenes* (9.0%), and *Alpha-hemolytic Streptococcus* (4.5%) in membranous obstruction (Fig. 3).

### Correlation between clinical characteristics and cytologic findings

**Figure 4:**
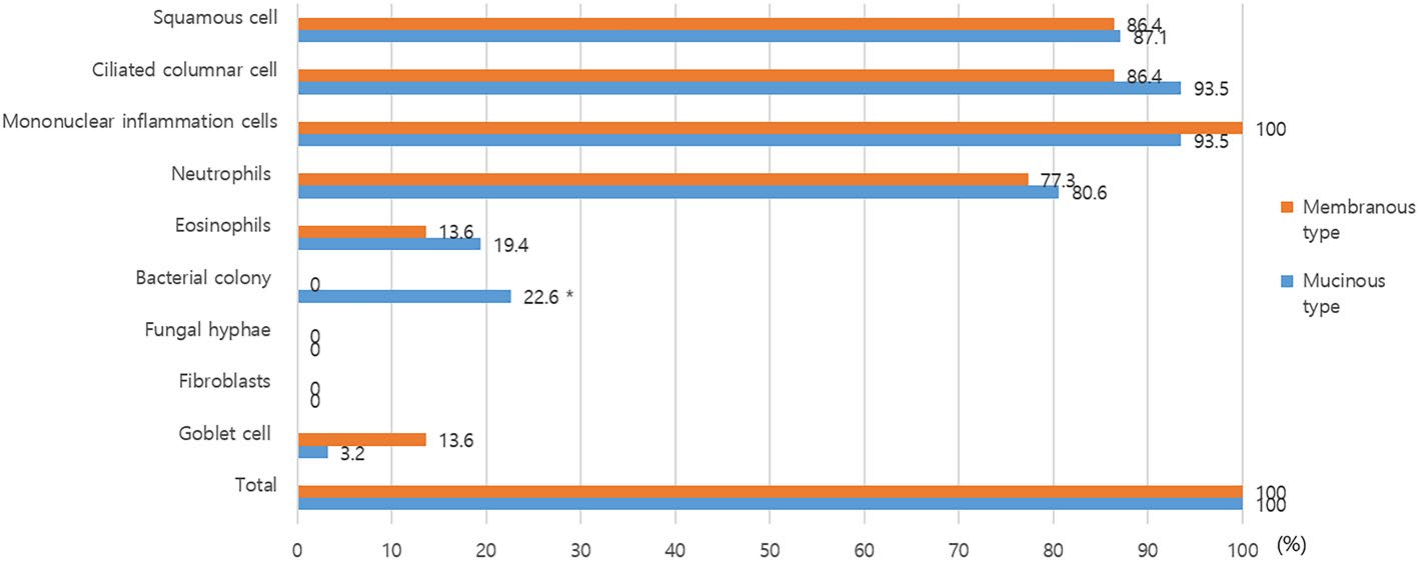
Comparison of cellular detection rate using the liquid-based thin prep cytology in the patients with mucinous type and membranous type (*p < 0.05).

The cellular detection rates from the LDS were as follows for mucinous obstruction versus membranous obstruction: squamous cells (87.1% vs. 86.4%), ciliated columnar cells (93.5% vs. 86.4%), mononuclear inflammatory cells (93.5% vs. 100%), neutrophils (80.6% vs. 77.3%), eosinophils (19.4% vs. 13.6%), and Goblet cells (3.2% vs. 13.6%). Bacterial colonies differed significantly between the two groups, with a higher rate in mucinous obstruction (22.6%) compared to membranous obstruction (0.0%) (p = 0.017). Fungal hyphae and fibroblasts were not detected in either group (Fig. 4). Based on the results from DCG, when classified according to the type of obstruction, there was no difference observed in cell detection for both squamous cells and ciliated columnar cells between the mucinous type and membranous type. However, when classified by the level of obstruction, the mucinous type was significantly more detected at the pre-sac level compared to the membranous type (Table 2). In the mucinous type, inflammatory cells containing mononuclear inflammatory cells, neutrophils, and eosinophils were predominantly detected at levels below the sac, corresponding to the primary change in obstructive type findings of DCG (Table 3).

**Table 2:** Comparison of mucinous type with membrane type based on detected epithelial cell types in liquid-based thin prep cytology study.

	Squamous cells			Ciliated columnar cells			Total	
	Mucinous type (N = 27)	Membranous type (N = 19)	*p-*value*	Mucinous type (N = 29)	Membranous type (N = 19)	*p-*value*	Mucinous type / Membranous type	*p*-value*
Type of obstruction								
Primary change	20	10	0.137	20	11	0.438	40/21	0.128
Secondary change	7	9		9	8		16/17	
Level of obstruction								
Pre-sac	22	5	< 0.001	7	8	0.002	29/13	< 0.001
Sac and post-sac	5	14		22	11		27/25	

*DCG* dacryocystography, *N* number.

*p-value < 0.05.

**Table 3:** Comparison of mucinous type with membrane type based on detected inflammatory cell types in liquid-based thin prep cytology study.

	Mononuclear inflammatory cells			Neutrophils			Eosinophils				
	Mucinous type (N = 30)	Membranous type (N = 22)	*p-*value*	Mucinous type (N = 25)	Membranous type (N = 17)	*p-*value*	Mucinous type (N = 6)	Membranous type (N = 3)	*p-*value*	Total	*P-*value*
Type of obstruction											
Primary change	20	11	0.231	18	8	0.106	5	1	0.157	63 (43/20)	0.016
Secondary change	10	11		7	9		1	2		40 (18/22)	
Level of obstruction											
Pre-sac	10	12	0.130	9	9	0.282	1	1	0.593	42 (20/22)	0.037
Sac and post-sac	20	10		16	8		5	2		61 (41/20)	

*DCG* dacryocystography, *N* number.

*p-value < 0.05.

## Discussion

PANDO is commonly diagnosed clinically using the canaliculus irrigation test in patients experiencing tearing.^11,12^ However, the trend of patency is not always correlated with anatomical stenosis.^13^ Similar to our previous study^14^, we observed a higher pass rate in cases of mucinous obstruction. Although the degree of NLD drainage dysfunction does not always correlate with the type or extent of obstruction, a combination of canaliculus irrigation tests and lacrimal imaging (DCG or dacryoscintigraphy) is used to confirm the severity of NLDO.^9,13,15–17^ However, the significant difference observed between the passed and not-passed rates suggests that mucinous obstruction involves distinct mechanisms or factors compared to structural blockage.

Compared to our previous study^7^, the modification of the cell harvesting technique enabled a significant enhancement in the cytologic harvest rate, rising from 84.8% to 100%. Additionally, when comparing mucinous obstruction with membranous obstruction in cytologic analysis, we discovered an intriguing finding wherein inflammatory cells were notably higher in mucinous obstruction. Hence, we recommend utilizing the highly productive modified cell harvesting method for investigating the PANDO environment.

To comprehend the fundamental etiopathogenesis of PANDO, the concept of the ‘lacriome’ was recently introduced, defining it as the collective microenvironments of the lacrimal drainage system.^18–21^ Metagenomic profiling of organisms isolated from PANDO patients commonly reveals the presence of bacteria, followed by viruses and archaea.^22^ In this study, we observed bacterial colonies in 22.6% of mucinous obstruction cases, but none in membranous obstruction, with a significant difference between the two groups. The bacterial colonies and bacterial detection do not always correlate. Bacterial detection confirms the type of bacteria through culture tests. However, bacterial colonies are deemed positive only when clusters of bacteria are observed under microscopic examination of cells obtained through our modified harvesting method. This finding is intriguing. The identified strains differed from those reported in other studies, which can be attributed to variation in race, region, and study subjects. The overall bacterial detection rate in our study was low, likely due to the collection of samples via saline irrigation rather than biopsy, with only bacterial colonies identified through microscopic examination of cells. S. aureus was the most commonly detected species; it is known to promote NLD inflammation and progress to continuous insularization and blockage.^23,24^ Our study aligns with this pattern, as *S. aureus* was the most frequently detected bacterium, and bacterial colonies were exclusively observed in cases of mucinous obstruction, suggesting that the pathogenesis of mucinous obstruction may be linked to bacteria-induced inflammation of the LDS.

The canaliculi are lined by nonkeratinized stratified squamous epithelium, while the lacrimal sac is lined by stratified columnar epithelium, containing goblet cells, glands, and cilia.^12,24–26^ In our study, squamous cells were detected in pre-sac obstruction in mucinous obstruction, while columnar cells were observed in sac and post-sac obstruction based on DCG findings. The presence of epithelial cells in mucinous obstruction correlated with the level of obstruction as identified in DCG findings, but not dacryoendoscopic findings. DCG aims to identify the site of initial occlusion of contrast media and diagnose the obstructed level, whereas dacryoendoscopy provides direct visualization of the LDS.^27^ Thus, the results obtained with these two methods may differ. Compared to membranous obstruction, mucinous obstruction had greater detection of inflammatory cells in the primary change of DCG findings, as well as in the sac and post-sac regions. This suggests that mucinous obstruction undergoes fewer secondary changes, such as beading or sac dilation, compared to membranous obstruction, and an inflammatory response occurs below the level of the lacrimal sac. Compared to the prior studies by the authors^10^, a commonality between the current study and the previous research is the focus on investigating the cytological characteristics of Primary Acquired Nasolacrimal Duct Obstruction (PANDO) and emphasizing the utility of the liquid-based thin prep cytology method. However, our study demonstrated a higher cytological detection rate using a modified technique, particularly highlighting distinct features in mucinous obstruction. Both studies offer profound insights into the pathophysiology of PANDO, with the current research particularly aiding in a better understanding of the cytological differences between mucinous and membranous obstructions.

Therefore, we propose that the pathogenesis of mucinous obstruction may involve an ascending inflammatory response originating from below the lacrimal sac.

Mucinous obstruction, confirmed at dacryoendoscopy, leads to tearing as either a single factor or in conjunction with LDS stenosis. To delve deeper into the underlying mechanisms and clinical implications of mucinous and membranous obstructions, our study investigated the cytological differences between these two types of obstructions. Mucinous obstruction exhibited a significantly higher detection rate of bacterial colonies and a tendency towards increased neutrophil detection compared to membranous obstruction. Such observations could potentially influence the choice of treatment strategies and offer valuable insights into the pathophysiology of these types of obstructions. The modified liquid-based thin prep cytology using a suction bottle proved effective at detecting cells and analyzing histopathological changes in the lacrimal passage of PANDO, particularly in cases of mucinous obstruction, without the need for invasive biopsies. These cytologic findings contribute to a better understanding of the etiology and changes occurring in the lacrimal passage of patients with mucinous obstruction in PANDO.

This study had several limitations. First, it was a retrospective review with a relatively small sample size. Second, the cytologic cell composition identified in this study could not be compared to the composition following symptom improvement. Despite these limitations, we introduced a newly modified liquid-based thin prep cytology method to analyze histopathological changes and cytological levels in PANDO, aiming to uncover the mechanism underlying mucinous obstruction.

In conclusion, there were no significant differences observed in the cellular compositions within the LDS between cases of mucinous and membranous obstructions. However, in instances of mucinous obstruction, squamous cells were notably more prevalent in the pre-sac level, while columnar cells were predominantly detected in the post-sac level. Furthermore, bacterial colonies and inflammatory cells were notably present in cases of mucinous obstruction. Therefore, we hypothesize that bacterial infection and resultant inflammation may contribute to the pathogenesis of mucinous-type lacrimal obstruction.

## Data Availability

The data supporting the main findings of the study are contained within the manuscript. Additional data are available from the corresponding author upon reasonable request.
